# Miscellaneous Intelligence

**Published:** 1800-05

**Authors:** 


### MISCELLANEOUS INTELLIGENCE.

---

To Dr. BRADLEY.

SIR,

THE inclosed advertisement will explain itself; but though I have renounced every idea of emolument, I doubt whether I shall have the audience I require. Mr. Davy and myself are going to publish on the gas, of which we have had so much and such agreeable experience in palsy.

I have had accounts from India of the successful use of acids in lues venerea and of the stability of the cures effected by them. At the Pneumatic Institution, and in private practice, they have proved strikingly efficacious. Perhaps, as a matter interesting to medical philosophy, you will allow me to add, that I want an assistant in physiological experiments; he must be dexterous in the use of the scalpel, and should be something of a draughts-man. Among the unsettled students of medicine, there must be individuals qualified for the undertaking; and I hope that their spirit of investigation will be sensible to this invitation. I had engaged a gentleman, whose whoſe talents were perfectly ſuited to my views; but an advantageous ſituation offering, I could not but give up my claims upon him.

I am, Sir,

Reſpectfully, your's,

*March* 12, 1800. THOMAS BEDDOES.

*Lectures on the Laws of Animal Nature, and of the Means of preſerving the Syſtem from Injury upon the moſt important Occaſions of common Life.*

At ſome convenient place in *Briſtol,* Dr. Beddoes propoſes to attempt a popular expoſition of the principles of the animal œconomy, with their application to the purpoſes of individual and domeſtic welfare, upon a plan widely different from that of any exiſting publication. For his opinion on the advantage of diſſeminating phyſiological information, he may refer to his *Lecture introductory* to Meſſrs. Bowles and Smith's Courſe of Anatomy; and an exemplification of the manner in which he thinks the ſubject ought to be treated, will be found in his *Eſſay on Conſumption.*

Heretofore an acquaintance with the cauſes of his perſonal condition has ſeldom been numbered among the *accompliſhments* of the ſcholar, or the *qualifications* with which the man of buſineſs is fitted out for ſucceſs in the world. Yet it will be confeſſed, that neither ſucceſs in buſineſs, nor proficiency in the ſciences, accounted *liberal,* are ſeparately ſufficient for rendering the condition of human life deſireable. And, in fact, to endeavour by any combination of theſe materials, to conſtruct a ſyſtem of perſonal happineſs, is to project an edifice which ſhall ſtand ſecure without a foundation.—Of a truth, ſo long and ſo generally neglected, a portion of the public, it is believed, begins to feel that degree of conviction which operates upon conduct. In this belief, the preſent opportunity of inſtruction is offered to thoſe who may be deſirous of it.

If it be allowed that the moral and phyſical attributes of human nature are inſeparable, perſons intereſted in the art of education will ſcarce require to be reminded of the value of that ſpecies of knowledge which the lectures, here announced, are intended to communicate.

They ought to prevent many of thoſe mortal bruiſes which travellers along the road of life give themſelves for want of knowing the quality and poſition of the objects in their way.

By preſenting a juſt eſtimate of *that art* to the operations of which almoſt every one is ſooner or later doomed to ſubmit, they ſhould afford ſome protection againſt groſs medical incapacity or fraud.

They ſhould reduce to their juſt value many of thoſe axioms that wander about the world concerning what is wholeſome or unwholeſome in diet or exerciſe:—axioms which the inſtinct of ſelf-preſervation impels men to take up; and upon which, however looſely adopted, they act with as full aſſurance as if they knew them to have the moſt ſolid foundation in phyſiological ſcience.

Numbers fall victims to their own impatience under illness, or to the wavering conduct of their friends. Frequently in the onset of dangerous diseases, people by suffering themselves to be amused by trifling domestic expedients, lose an opportunity which no medical skill can retrieve. Upon these evils the prevalence of juster ideas would act as a check. Nor is it paradoxical to suppose that the mortality among infants would be smaller, and debility of constitution at all periods of life more rare, if parents (however instructed in other things) were not in common nearly upon a level with nurses in that which it so much imports them to possess—an acquaintance with the powers that operate to the injury or advantage, the destruction or preservation, of the objects of their affection.

The Author further hopes (if he may repeat his own words) to contribute towards preventing the "ignorant from tampering with the sick; towards promoting the ascendancy of science over intrigue; alluring curiosity from the pernicious frivolities of literature, and elevating the conceptions of men to the level of their highest interests."

As the whole course will be connected, the tickets will not be transferable—The number of lectures cannot be determined beforehand—But that there may be little chance of exclusion by reason of narrow circumstances, the subscription is fixed at ONE GUINEA—The lectures will be calculated for both sexes and different ages—They will be delivered in the evening, and commence sometime in April next—probably near the middle of the month—provided fifty persons shall have entered their names by the 31st of March. This condition is indispensable. Without a tolerably numerous audience, the author presumes he could bestow his time in a manner more advantageous to the public.

Subscriptions received by Mr. SHEPPARD, Bookseller, *opposite the Exchange*, with whom conditions for printing a SYLLABUS may be seen.

*Rodney-Place, Clifton, March* 3, 1800.

At the MEDICAL PNEUMATIC INSTITUTION, *Dowry Square, Hotwells*, Out-patients continue to be received every Sunday and Wednesday morning.—It is particularly wished that the consumptive and those troubled with obstinate venereal symptoms which have resisted mercury, as also persons who have lost the use of their limbs from the palsy, would apply.—Two or three paralytic patients could now be received into the house.

---

In Dr. Christ. W. HUFELAND's Observations on Natural and Inoculated Small Pox, &c. 1798, the celebrated author informs us, that he had found the tincture of the seeds of thorn apples to be, in many cases, superior, as a narcotic remedy, to opium, as it neither heats, nor causes costiveness; and, that he had cured with it *all* obstinate affections of the mind and convulsive symptoms.

 The

The feed of the thorn apple has not hitherto been ufed in medicine; but if experience fhould prove it worthy of the encomium beftowed upon it by Hufeland, it certainly deferves to be admitted to a place in the *materia medica,* inftead of the plant.

---

Profeffor Blumenback, in letter to a friend (Dr. Afh) in this country, fays, that a fecret is now making as much noife in Pruffia as the Cow-pock in England.

A Profeffor Reich in Erlangen, fays he has difcovered two medicines, one internal, the other external; by means of which, all the dangers of acute difeafes may be warded off, as it were, in a moment, only the ufe of them muft not be deferred too long.—Thefe medicines do not make a fudden or complete cure; but by means of them, all danger is fo far removed in twelve hours, that it may be fecurely prognofticated that the life of the patient is fafe. He has offered to communicate his fecret to every practitioner for a moderate douceur; and he his now ordered by the King to be examined before a committee of phyficians at Berlin.

Mr. Blumenback adds, that Profeffor Reich is a plain, fenfible man, and not the leaft inclined to quackery. Profeffor Sprengel fancies he has found thefe two medicines in the works of an old Arabian Phyfician.

---

Mons. Chambon, a phyfician of eminence in Paris, has publifhed a new edition of his works, on the Difeafes of Women and Children, with confiderable additions; they now appear in ten volumes, 8vo. The two firft volumes contain the Difeafes of Infants; the third and fourth treat on the Complaints and the Management of Girls at Puberty; the fifth and fixth contain the Difeafes attendant on Pregnancy; the feventh and eighth treat on the Difeafes incident to Lying-in Women; and the two laft contain Obfervations on Chronic Difeafes, and particularly the treatment of thofe occuring about the Ceffation of the Menftrual Evacuation.

---

Projet d'inftitution d'un hofpice anti-hydrophobique dans la capitale, par le C. Antoine-Alexis, Cadet-le-Vaux.

The idea of an hofpital for patients in hydrophobia is about to be realized, and the author propofes to fet apart one or two wards for the obfervation and treatment of mad dogs, and dogs faid to be mad, in order to remove the anxiety from patients who have unfortunately been bitten; when dogs are fo immediately deftroyed, it becomes impoffible to afcertain whether they have been really mad or not. This plan will alfo afcertain the propriety or the folly of trufting to the effects of certain *infallible* medicines for the prevention and cure of hydrophobia.

---

Dr. Smith, prefident of the Linnean Society, has juft publifhed two volumes of his Flora Britannica, which the botanical world have been anxioufly expecting fome time; we underftand two more volumes will complete the work.

In the Philosophical Transactions, Jan. 9, 1800, Mr. Carlisle has communicated the discovery of a peculiar arrangement in the arteries of certain slow moving animals. The result of his examination has been this: In the lemur tardigradus, the axillary and iliac arteries, instead of ramifying in the usual arborescent form, suddenly, at their entrance into the limbs, divide into a number of equal sized cylinders, which occasionally anastomose with each other. These are exclusively distributed to the muscles, while the branches which supply other parts pass off in the usual manner.

A similar peculiarity has been found also to exist in the bradypi. In the tridactylus, however, the branches have considerably greater communication with each other than in the lemur tardigradus, and their number is considerably greater. In the bradypus didactylus, the variation from the usual mode of distribution is less extensive, the brachial artery being much less divided, and the iliac artery afterwards giving off some arborescent branches; but, it is to be remarked, that the motions of this species of sloth are universally allowed to be quicker than in the tridactylus. An examination of the lemur loris afforded the same results.

Mr. Carlisle promises to prosecute these researches, which will, very probably, lead to a complete elucidation of muscular motion.

---

Mr. James Christie intends publishing, shortly, An Essay on the Nature and Cure of an Epidemic Fever, combined more or less with Pneumonia, as it has appeared of late among the troops of the line.

